# Responding to Life Itself: A Proposed Understanding of Domain, Goal and Interventions for Chaplaincy in a Secular Age

**DOI:** 10.1177/15423050241296785

**Published:** 2024-11-05

**Authors:** Job Smit, Carmen Schuhmann, Annelieke Damen

**Affiliations:** Faculty of Theology and Religion, VU Amsterdam, the Netherlands; 36513 Department of Humanist Chaplaincy Studies for a Plural Society, University of Humanistic Studies, Utrecht, the Netherlands

**Keywords:** Spirituality, meaning, method, secular age, chaplaincy, spiritual care

## Abstract

In this article, we present a model of chaplaincy in a secular age which includes, in one coherent system: domain, goal and an intervention pathway. The domain is presented as the process of “responding to life itself”. A corresponding goal of chaplaincy is considered in the context of “existential well-being”. This goal can be achieved through the proposed “The Ritual Bath Model” based on these new defined concepts.

## Introduction

In Western societies, religion-based professional chaplaincy has existed for over a century as a specific type of care in the public domain (Cadge, 2019). Initially, this care was inspired by Christian faith traditions, but due to processes of secularization, Muslim, Buddhist, Hindu, humanist, pagan, and (at least in the Netherlands) so-called ‘unaffiliated’ chaplains have joined the field. Charles Taylor (2007) describes secularization in Western societies as “a move from a society where belief in God is unchallenged and indeed, unproblematic, to one in which it is understood to be one option among others” (p. 3). In this sense, the spiritual landscapes of Western societies have become more diverse, as people integrate an increasing variety of religious and nonreligious belief systems in their worldviews (Ai & McCormick, 2010; Liefbroer & Berghuijs, 2019).

The changes in the landscape of worldview, religion and spirituality have impacted the chaplaincy profession regarding at least three features. Firstly, questions arose about the domain of chaplaincy care. An understanding of the domain in traditional religious terms did not include chaplains from a diversity of religious and nonreligious backgrounds, nor the wide range of worldview backgrounds of their clients (Nolan & MacLaren, 2021). Secondly, questions arose regarding the ‘goods’ or goals chaplains aspire to in their practices, following Alisdair MacIntyre's line of thought that “every activity, every inquiry, and every practice aims at some good” (2007, p. 173). The goals were traditionally understood in religious terms - like being a better Christian; an understanding that no longer reflected the diverse practices of chaplains, nor the spiritual needs of their clients (Schuhmann & Damen, 2018). Finally, questions arose whether the traditional chaplaincy interventions could still accommodate clients’ different worldviews. (Nolan & MacLaren, 2021).

In response, several authors have pointed to the need to develop new overarching theoretical underpinnings for the profession of chaplaincy (Harding et al., 2008; Kevern & McSherry, 2015; Lasair, 2019; Thorstenson, 2012; Zock, 2008), and inclusive interventions (Jacobs, 2021; Nolan, 2021; Wierstra et al., 2023). For example, Kevern & McSherry have argued that “in a society where the proportion of people who report themselves as of no religion is increasing … there is a need for a discourse on chaplaincy which preserves its core value but speaks to people of all religions and none” (2015, p. 49. cf. Cadge & Rambo, 2022). Also, Liefbroer and colleagues concluded that “spiritual care interventions that are tailored to the specific needs of palliative care patients in a secularized and religiously pluralized context, are remarkably scarce (2022, p. 1).

In recent decades, several attempts have been made to develop a broad professional profile for chaplains (Association of Professional Chaplains et al., 2001; Den Toom et al., 2021; Cobb, 2004; Schipani, 2013; Schuhmann & Damen, 2018). Attempts have been made to identify generic goals for chaplaincy care (Visser et al., 2022), or describe generic interventions (Kruizinga et al., 2013; Wierstra et al., 2023). None of those attempts, however, have connected the chaplaincy domain with chaplaincy goals and chaplaincy interventions in one overarching framework that shows how these dimensions interlock. In this article, we would like to present such a model. We explore the following three questions: (1) What generic language can describe the domain of the chaplaincy profession? (2) What generic goal can encompass diverse chaplaincy practices? (3) Which generic method can support clients from different worldview backgrounds?

This exploration is based on the PhD thesis of the first author (Smit, 2015). By formulating a comprehensive model, we hope to contribute to discussions among chaplains on their collective professional self-understanding, and to support them in confidently accounting for their professional activities within a secular context.

## The Domain of Chaplaincy: Responding to Life Itself

The domain of chaplaincy is nowadays generally understood in terms of spirituality: chaplains are professionals who provide care in the spiritual domain. Associating chaplaincy with spirituality does not, however, unequivocally answer the question of what is meant by the term in a secular age. On the one hand, there is a development towards perceiving spiritual care as a joint effort of various care professionals, not as the exclusive task of chaplains (Puchalski et al., 2014). On the other hand, spirituality is a notoriously hazy concept that is defined in various, sometimes contradictory, ways (Hill et al., 2000; Jastrzębski, 2022; Walton, 2012, 2013). The much used consensus definition by Puchalski et al. (2014) conceives spirituality as a process: “Spirituality is the aspect of humanity that refers to the way individuals seek and express meaning and purpose and the way they experience their connectedness to the moment, to self, to others, to nature, and to the significant or sacred” (p. 643). This definition is comprehensive but also complex. The elements of this definition are heterogenous (and are sometimes contested themselves), and the relation of those elements to each other is unclear. Because it is a consensus definition, it lacks a philosophical underpinning. Therefore it is difficult to grasp the underlying and integrating idea that connects the various elements. For theoretical and communicative purposes, it would be helpful to look for a concept which can more easily be grasped and intuitively understood.

In this article, we elaborate on the proposition by the first author (JS) to use the term ‘responding to life itself’ in order to denote the spiritual process that constitutes the domain of chaplaincy. Without aiming for a full overview, we will briefly indicate how various authors from various disciplines support this idea. Sometimes this process is referred to in terms of a search for meaning (Pargament, 2001; cf. the recent volume, edited by Cadge & Rambo, 2022, in which meaning-making is seen as central in chaplains’ work). First we turn to the tradition of Logotherapy initiated by Viktor Frankl. Existential psychologist Wong (2012), following Frankl (2014) and Fabry (1998), uses the term meaning when pointing out that people have to face the fundamental fact that they exist in this world, and that this instigates a quest for meaning. No one can evade ‘the call of meaning’, as Fabry puts it. This echoes Frankl's concept of the ‘demand quality of life’ meaning that ‘life is the questioner and the human person is called to respond to life in the most meaningful way’ (Graber (2004) p. 36–37; Frankl (2014). The theologian Fowler uses the term ‘faith’ - which he interprets as a universal anthropological phenomenon - in relation to processes of responding to the ‘call of life itself’: “Faith is a response to action and being that precedes and transcends us…faith is a human phenomenon, an apparently generic consequence of the universal burden of finding or making meaning.” (1981, p. 33). He also highlights the behavioral-ethical character of this response: “…Faith is a person's or group's way of moving into the force field of life.” (p. 4). Thirdly he stresses the holistic character of this response: “Faith.is not a dimension of life, a compartmentalized specialty. Faith is an orientation of the total person, giving purpose and goal to one's hopes and strivings, thoughts and actions’’ (p. 14).

In the field of philosophy, we find similar ideas. The Canadian philosopher Charles Taylor (1989), for instance, elaborates on the notion that we are on a quest for meaning in response to an existential call. He states that, in living our lives, we respond to the call of existential questions, even when we are not actively reflecting on them. The German sociologist Hartmut Rosa (2019) builds on ideas by Taylor when explaining existential processes in terms of a longing for ‘resonance’: “Resonance presupposes the existence of that which is non-assimilable, foreign, and even mute; only on this basis can an Other be heard and respond without this response being a mere echo or repetition of oneself” (p. 185). This echoes a central thought in the work of the French philosopher Emmanuel Levinas (1969) about how ‘the call of life itself’ may be experienced in the form of an ethical appeal emanating from the human ‘face’ of the Other, which speaks to me: know me, recognize me, do not destroy me. The confrontation with the Other strikes us, invades us, and calls for a response - it incites motion and action. In Rosa's view, ‘the call of life itself’ is not primarily understood in terms of (rational) questions that life poses to us, but rather in terms of being touched or affected by the world. Rosa also stresses the fact that resonance is bodily mediated.

The fundamental call that life itself poses to us can be formulated as “I am here, the world is here - what is next, how am I going to live my life in this world?” This call is not primarily to be understood as the call(s) we experience *in* life, but as the fundamental call which emanates from the very *fact* of our existence in this world. That is what the additional term ‘itself’ indicates. The term implies contingency and transcendence. It implies contingency in the sense that life is a given: it might not have been there at all or have been there in a different way. Worldviews comprise a view of how to understand and respond to the ‘givenness’ of life- a view which may or may not include the notion of a ‘giver’. Furthermore, life ‘as a whole’ implies transcendence in the sense of the possibility of relating this ‘whole’ to something else: a transcendent reality, a personal God, an ultimate value or a driving force. We understand this to be a common theme present in all religious and nonreligious worldviews which warrants a specific professional domain.

### The Responding Process

**Figure 1.**
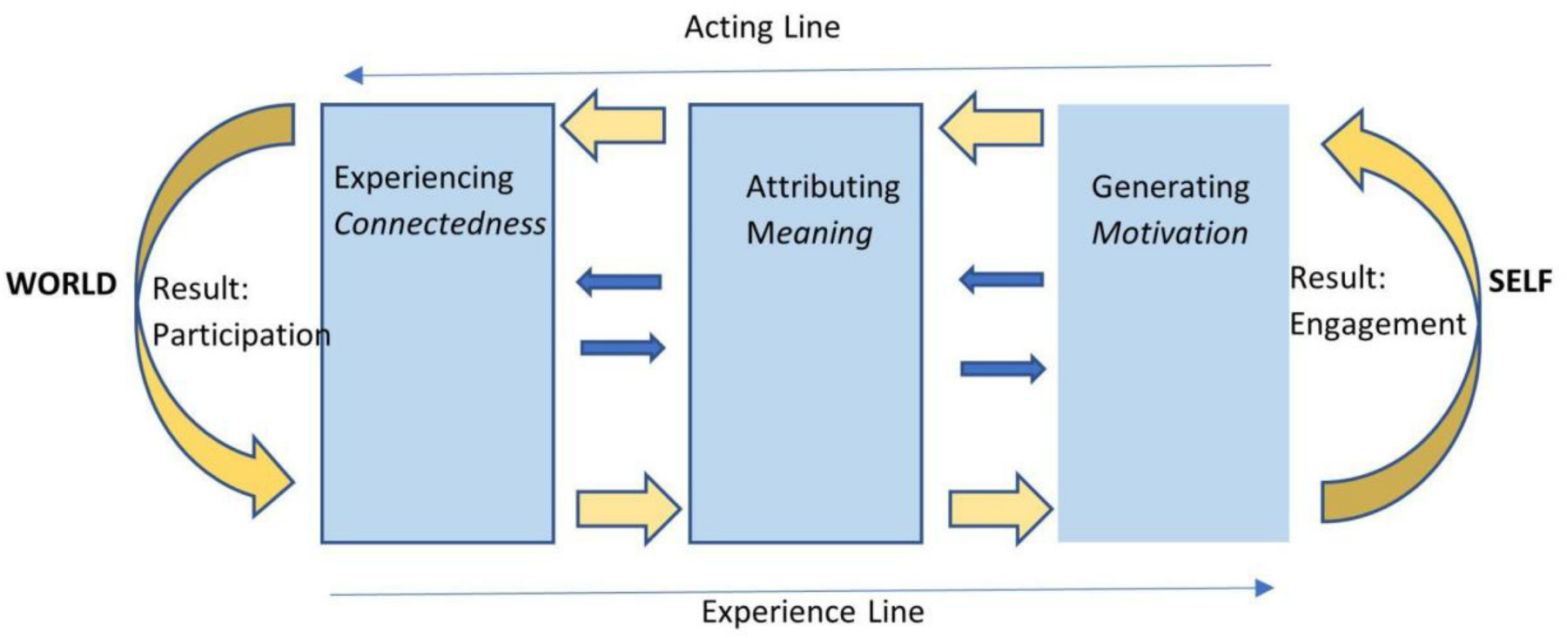
The Responding Process.

As the domain of chaplaincy is conceived in terms of a process, we must ask ourselves what this process looks like. The first author of this article (JS) proposed a theory-based model that starts from the observation that the term ‘meaning’ – the dominant domain description of chaplaincy in the Netherlands – is used in three different discourses: pre-modern, hermeneutical, and psychological. First, in pre-modern discourse, meaning refers to finding one's appropriate place in the cosmological order. We translate the pre-modern notion ‘appropriate place in the cosmological order’ for the modern mindset into the notion of ‘experience of connectedness’. Second, since in modernity a supposed order is no longer self-evident, ‘meaning’ has come to refer to a hermeneutical, mainly cognitive, process of creating order by attributing meaning to events that at first seem to be contingent. Third, in the twentieth century, psychiatrist Frankl initiated an understanding of meaning as a strong motivational force in life, citing Friedrich Nietzsche: “He who has a Why to live for can bear almost any How.” (2014, p. 72). These three modi of ‘meaning’ refer to three heterogenous life dimensions and life dynamics. The diagram below shows how these three modi of meaning can be integrated in a comprehensive conceptual model of the process of responding to life itself (see Figure 1).

In this model, the responding process is conceptualized as a circular movement in which ‘self’ and ‘world’ interact. Thereby it is of a fundamental relational nature which transcends the mere psychological and cognitive dimensions. We begin our explanation with the left column, where the responding process starts at a pre-reflective, affective level, in which we experience connectedness with the ‘world’ (personal and non personal). It is within this sense of connectedness that we discern the call of life (Cf. Rosa, 2019). Such an experience of connectedness may give rise to the thought “this is how life may be!”, which essentially is a cognitive-hermeneutical, (re)evaluation of life. It is here that the hermeneutical process of meaning attribution starts. This is symbolized in the middle column. That, in turn, generates a motivation to act in line with the evaluation (‘when this is what life means to me, then I want to/have to act so and so’). This is symbolized in the right column. One can say that within the sphere of the first column, the ‘call of life’ is experienced in a pre-reflective way, while the call of life may be consciously articulated in the right column.

When we look at the large arrows, the movement from left to right represents a process which generates engagement with life: people want to take responsibility for their lives. This is a pivotal moment in the circular responding process. The arrows from the right to the left depict the acting phase in the process. When people act in the world, they attribute meaning to their actions, which may involve clarifying and justifying their worldview and how they aim to affect the world through their actions. This eventually deepens the experience of being connected with the world. In the diagram we then return to the first pillar of experiencing connectedness. The completion of this movement results in participation in the world: people feel that they are part of and at home in the world. The whole process is conceived as a fundamental ongoing process which colors all other life processes.

Several arguments support the relevance and usefulness of this conceptual model of the meaning process for chaplaincy. First, in the model, the heterogeneous factors of the responding process - a process that goes by various names - are taken into account: the relational, the hermeneutical and the motivational. Second, the model describes how those factors interlock in a process that has an intrinsic functionality, as it leads to engagement with life on the one hand, and participation in the world on the other hand. Third, the model helps to elaborate on the notion of ‘spiritual need’. Spiritual needs appear as disturbances of the responding process which may occur within any or each of the three modi of meaning, or when the flow between the modi is blocked at some point. Fourth, the model structures potential chaplaincy interventions. Relational, hermeneutical and motivational (spiritual) interventions (in diagram: all the three pillars) can all contribute to keeping the responding process going according to their own structure and dynamics. Fifth, the model takes into account that the responding process is not only about how we *experience* life (arrows from left to right in the diagram) but also about how we *act* in life (arrows from right to left).

## The Good or Goal of Chaplaincy

The next step in formulating a comprehensive model is to formulate a generic goal of chaplaincy. There is little consensus within the profession about relevant chaplaincy goals, and not every chaplain is familiar with thinking in terms of goals of their actual practice (Visser et al., 2022). The profession has been significantly influenced by the person-centered approach, in which the agenda of the contact is left in the hands of those being served. Many stress the non-protocolized nature of the spiritual contacts (Cadge, 2023). Working with goals is often misunderstood as working with a predetermined plan to achieve certain outcomes, opposed to, for example, a focus on forging a healing relationship (Damen et al., 2020). We would like to propose another understanding of a ‘goal’ that is in line with chaplaincy values. For that, we turn to the philosopher Alisdair MacIntyre who says that a practice refers to

“…any coherent and complex form of socially established cooperative human activity through which goods internal to that form of activity are realized in the course of trying to achieve those standards of excellence which are appropriate to, and partially definitive of, that form of activity…” (MacIntyre, 2007, p. 218). In this understanding, goals are not instrumental targets but the ‘goods’ that a practice aims for (MacIntyre, 2007). We would argue that chaplains in their practice aim at bringing about some change for the better for their clients, which can be understood as striving towards certain goods. Where a physician seeks to achieve physical well-being, a psychologist emotional well-being, a social worker social well-being, the chaplain seeks to achieve well-being with regard to the processes of responding to life itself. This generic goal of chaplaincy may be called ‘existential well-being’.

In line with the model of the responding process, existential well-being consists of four levels:

1. Engagement and participation in life. Following the model of the responding process, the most encompassing goal is *engagement with and participation in life.* One can speak of existential well-being when responding to life itself generates engagement and participation in life: I *want* to go on with my life because I have hope.
2. Relational-ontological security. The modus of ‘experience of connectedness’ gives rise to the goal of *relational-ontological security*. Here existential well-being comprises a sense of basic trust in the (O)other (be it people, the world as a hospitable place, and/or a transcendent being): I *may* go on because I receive the space and recognition from others or the Other to do so.
3. Worldview vitality and plausibility. Based on the notion of meaning attribution existential well-being means: having a *worldview* from which one derives strength, motivation, and direction in one's current situation *(vitality),* and which is sufficiently embedded in one's socio-cultural context *(plausibility*): I *can* go on because I have sufficient sources of strength and competences.
4. Spiritual congruence. On the deepest level, one can speak of existential well-being when a person shapes his/her life in accordance with the experienced call of life itself: ‘am I the person I am called to be?’ When this succeeds, we call this *spiritual congruence*: I *dare* to go on because I have confidence.

The four levels are structured in a hierarchy. The most visible aspect of someone's existential well-being is engagement and participation in life. Less apparent is the relational-ontological security a person experiences. On a still deeper level, one can get to know the vitality and plausibility of someone's worldview. At the most intimate level, one can become aware of someone's spiritual congruence. With the emphasis on spiritual congruence as the deepest level of existential well-being, we want to stress that existential well-being is not to be equated with absence of suffering. On the contrary: it is well known that people may consciously choose to endure hardships in order to fulfill their calling. Spiritual congruence may be valued more than physical comfort or psychological peace: one can be happy in unhappiness, feel good in the midst of suffering, or feel blessed in times of deprivation. Existential well-being can therefore also be understood as a critical notion: it is not primarily a contribution to well-being or happiness at large, but rather *defines* them. It is not a dimension of well-being next to other dimensions of well-being, but underlies and colors all other dimensions of well-being. Finally, we do not understand the fourth level as the only goal of all chaplaincy interventions. Chaplains move between the different levels in their care practices. On every level chaplaincy support may take on a different structure and form.

## An Intervention Pathway for Chaplaincy: the Ritual Bath Model

Physicians and other care professionals often follow the pathway of ‘anamnesis - diagnosis - therapy - control’. Joan Tronto (1993) developed a pathway for care: ‘caring about - taking care of - providing care - responding’. In a similar vein, we will now propose a generic chaplaincy intervention pathway in line with the above goal of chaplaincy care which can structure and integrate all kinds of interventions: relational, hermeneutical (narrative) and spiritual.

**Figure 2.**
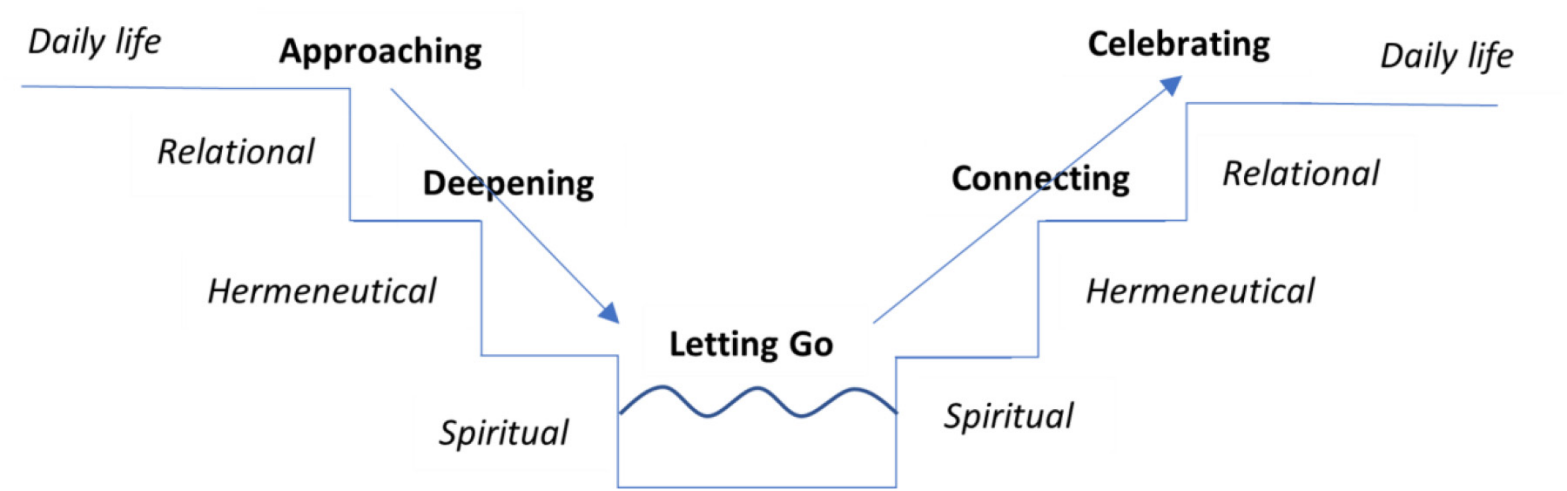
The Ritual Bath Model.

The pathway is based on the theoretical reflections above (see supplementary figure 1),^1^ and a small empirical study with eight Dutch chaplains from different chaplaincy sectors (healthcare, army, penitentiary, elderly care, revalidation, and psychiatry) and worldview orientations (Protestant, Catholic, and humanist). The chaplains were asked about the aims of their care, and how they sought to realize them. (i.e., their method). Subsequently, their responses were compared to transcripts of their conversations. The majority of the respondents formulated the goal of their practice in relational terms: they wanted their clients to be heard, seen, and acknowledged. However, they all added a quality criterion: they wanted their clients to be *really* heard etc*.* Remarkable was their use of the root ‘-deep’: they wanted to have *deep* contacts, they sought a *deepening* of the conversation, they wanted to go ‘in *depth’,* etc. In short: they wanted their clients to be seen, heard and acknowledged *in their deepest motivations, inspirations and aspirations.* These findings inspired the Ritual Bath Model, based upon interaction between the surface and the depth of life (see Figure 2). To be clear, ‘ritual’ includes non religious professionals, as also non religious professionals are acquainted with rituals.

The Ritual Bath Model may help chaplains to maintain a focus on the goal of chaplaincy care. It should be understood as an ideal-typical intervention pathway which may be followed in (part of) a conversation, multiple conversations, or group activities. The overall metaphor of the pathway is the cleansing of a ritual bath: with respect for the client's autonomy. The chaplain descends with the client step by step into the deepest level of life, that is the spiritual level (commonly called ‘heart’ or ‘soul’) where one is touched by the call of life. Subsequently, the chaplain and the client ascend step by step to the surface of daily life again.

The pathway starts on the daily level in which the chaplain and the client meet each other and become acquainted with each other's personal space (*approaching*). A *deepening* of the contact is facilitated by the chaplain through a conjunction of relational and hermeneutical interventions. At some point, a person's deepest motivations, inspirations and aspirations may come into view. On that spiritual level, the task of the chaplain is *to let go*, to not want or do anything, but just be an accepting and loving witness. A spiritual disclosure may happen: something is seen, heard or experienced that has not occurred until now (Ramsey, 1957). Signs of the disclosure can be discerned: a silence, moments of introspection, tears, an inner search for words or an unexpected reaction (cf. Sauer Bredvik, 2021). After that, the way up to the surface begins, the phase of *connecting* to daily life. The first action of the ascending phase is to try to find words or other expressions for what happened on the spiritual level in order to make the experience communicable and fruitful, helping to continue one's life in a meaningful and more fulfilling way. Because experiences at the spiritual level are often beyond rational understanding, finding words for these experiences may predominantly be done by metaphor and symbol or by exploring the effects of the experiences on the client. Then a journey of discovery starts into how to integrate the disclosed insight into daily life. The last phase of The Ritual Bath Model is *celebrating* the contact between the chaplain and the client. They evaluate the contact: what has been given and received, and how is it valued? The contact ends with religious or nonreligious good wishes for the continuation of life, adequate for the situation (a competence to be practiced).

The ideal-typical nature of the pathway does not mean that chaplaincy has only been helpful when the spiritual level has been reached. Often people don not want to ‘take a bath’. Perhaps they will do it some other time or with another person. They might ‘jump’ to the other side before the spiritual level has been reached, which is fine because, from a spiritual perspective, every kind of deepening of the contact is fruitful.

On the basis of this pathway some remarks on the specific role and expertise of the chaplain can be made. In the ‘descending phase’, the role of the chaplain is mainly explorative. In the ‘ascending phase’, chaplains may also take an advisory role. Relational competencies are important throughout the whole process, but they are pivotal in the approaching phase. In the deepening phase, meaning attribution is key. Here, narrative theory is of methodical relevance. It should also be noted that this deepening process is placed within the context of a human encounter and therefore is performed in a dialogical modus. No one has got the definitive answers when it comes to the call of life, so searching for answers has to be a joint effort. In order to contribute to this dialogical process, the chaplain should not only have knowledge of the formal aspects of meaning attribution, but should also be acquainted with the substantive aspects of meaning making found in religious and nonreligious worldview traditions. Worldview and religious insights can prompt, suggest, confirm or elicit ways of responding to life itself, because responding to life itself is the very heart of worldviews and religions.

## Discussion

In this article, we presented a comprehensive model of chaplaincy in a secular age comprising the three interrelated features of the domain, goals, and interventions of the chaplaincy profession. We proposed to understand the domain of chaplaincy practice using vocabulary based on the notion of ‘responding to life itself’ which denotes the spiritual process arising from experiencing the ‘call of life’. This vocabulary can be related to all kinds of worldview traditions (religious and nonreligious), as it comprises both contingency - life itself as a ‘given’- and transcendence - the possibility of relating to life as a whole. Chaplaincy can then be understood as providing support concerning the process of responding to life itself. We described the overarching goal of this support in terms of ‘existential well-being’, explaining that existential well-being can be experienced at various levels, in line with the various elements of the process of responding to life itself. In this view, existential well-being comprises engagement and participation in life based upon relational-ontological security, worldview vitality and plausibility and spiritual congruence. Finally we presented a generic chaplaincy intervention pathway towards existential well-being which can serve as a framework for more specific interventions: The Ritual Bath Model.

This comprehensive model is worldview inclusive: all worldviews offer interpretations of life, whether or not this involves a notion of God. The model is also methodically inclusive. The intervention pathway accommodates insights from different theories - relational, psychological, hermeneutical, therapeutic and ethical. At the same time the model structures the application of these theories. Finally, the model is context inclusive. We are at all times caught up in a responding process - the question ‘how am I going to lead my life’ is not restricted to specific contexts or situations.

An aim of developing the model was to support chaplains to account for their practices in secular contexts, both to other professionals or managers in health care, and to themselves. Explaining the aim of their practices in terms of existential well-being allows them to align with well-being discourses used by other professionals while also emphasizing what is their specific contribution. Given the critical potential of the notion of existential well-being, adopting the term existential well-being also allows chaplains to keep a critical position when it comes to dominant discourses of well-being in organizations and society. We hope that the model may also help chaplains when it comes to evaluating their own practices. The idea is the following: if, as a result of chaplaincy care, clients move closer to the goal of existential well-being - at any of the four levels that were described - chaplains have done a ‘good’ job. Chaplains may use the model as a lens for assessing what changes chaplaincy care has brought about when they reflect on their own practices or ask clients about their experience of the care they provided. The model may also be helpful when it comes to outcome studies into chaplaincy. Damen et al. (2020) have argued that outcome studies should more often focus on characteristic chaplaincy outcomes, that is, outcomes that are related to characteristic chaplaincy goods. In terms of the model, the crucial question then is what outcomes are related to the different levels of existential well-being.

A limitation of the comprehensive model is that it has not yet been empirically tested. The model is based on extensive literature review, reflection and a small empirical study, but not on experiences of chaplains using the model. Another limitation of this article is that, for lack of space, we could not make extensive comparisons with other descriptions of the domain, goal and interventions of chaplaincy. It requires further reflection and research to substantiate the claim that the common terms like ‘meaning’ and ‘spirituality’ really can be understood as aspects of the overarching concept of the ‘responding process’ and thereby obtain a more focused meaning. Finally, future empirical research could reveal the sustainability of this model in various contexts and for various worldview traditions. As the Ritual Bath Model is being used by chaplains, chaplaincy students, supervisors and chaplaincy teachers in the Netherlands, this would be a logical next step for future research.

## Supplemental Material

sj-docx-1-pcc-10.1177_15423050241296785 - Supplemental material for Responding to Life Itself: A Proposed Understanding of Domain, Goal and Interventions for Chaplaincy in a Secular Age Supplemental material, sj-docx-1-pcc-10.1177_15423050241296785 for Responding to Life Itself: A Proposed Understanding of Domain, Goal and Interventions for Chaplaincy in a Secular Age by Job Smit, Carmen Schuhmann and Annelieke Damen in Journal of Pastoral Care & Counseling

## Notes

1. With regard to the theoretical reflections, a coherence with the responding process model may be observed. In diagram language: the lower arrows from left to right in the process model correspond to the downward movement in the Ritual Bath Model. The upper arrows from right to left correspond to the upward movement in the Ritual Bath Model. The spiritual turning point in the responding model (where engagement with life emerges) is symbolized at the bottom of the Ritual Bath Model. The relational and hermeneutic factors both play a role in the deepening and the connecting phase. The effects of engagement and participation are situated in the celebration phase and the life thereafter.
