# Supplementary figures and images for: Responding to Life Itself: A Proposed Understanding of Domain, Goal and Interventions for Chaplaincy in a Secular Age
Source: J Pastoral Care Counsel. 2024 Nov 5;78(4):188–95. doi: 10.1177/15423050241296785 (PMC11616222; doi:10.1177/15423050241296785)

**Supplementary figure 1. The anchoring of the Ritual Bath Model in the Responding Process Model**

**
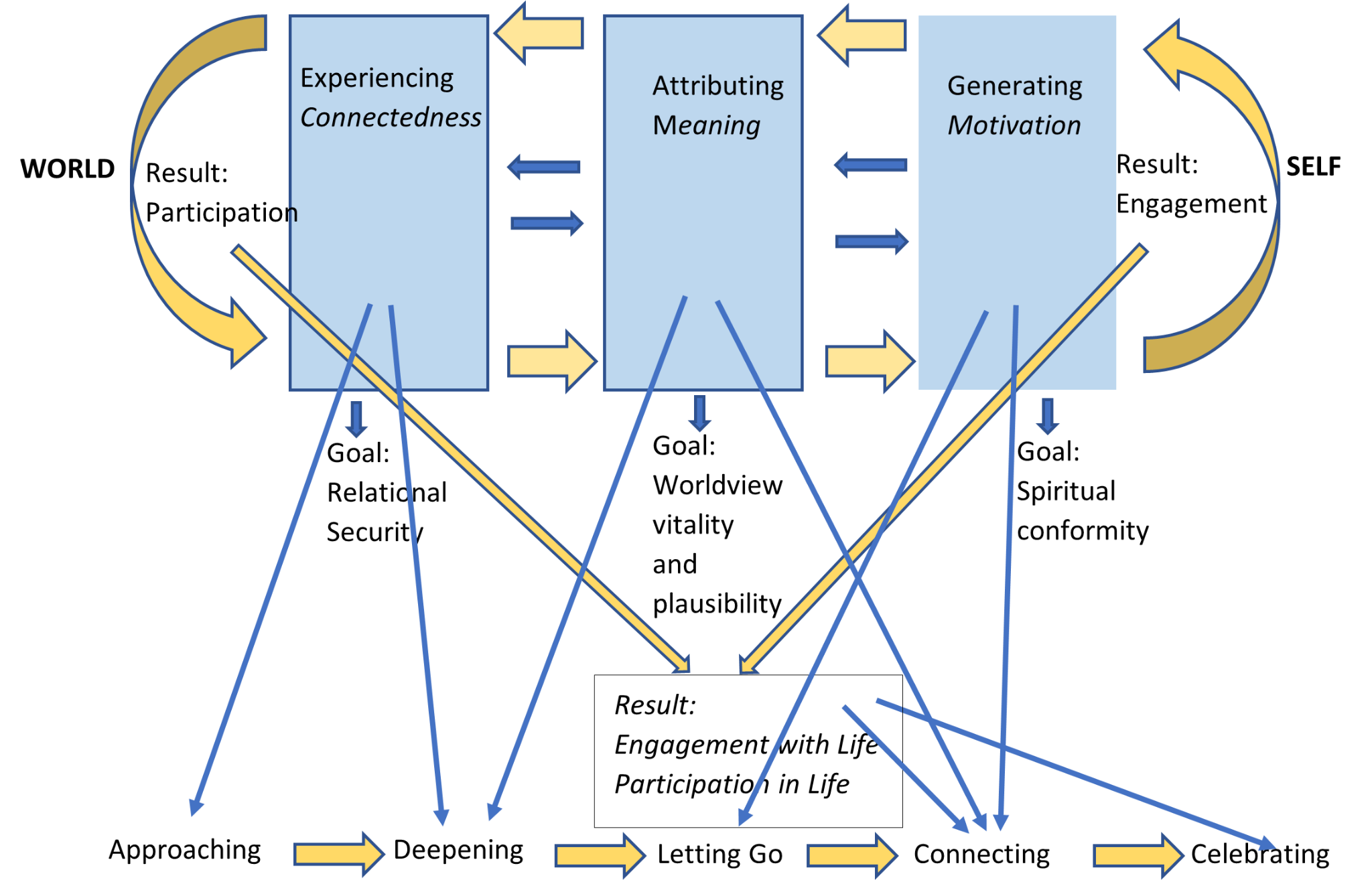
**

Supplement: sj-docx-1-pcc-10.1177_15423050241296785 - Supplemental material for Responding to Life Itself: A Proposed Understanding of Domain, Goal and Interventions for Chaplaincy in a Secular Age [file sj-docx-1-pcc-10.1177_15423050241296785.docx]
